# High-Fidelity Inhomogeneous Ground Clutter Simulation of Airborne Phased Array PD Radar Aided by Digital Elevation Model and Digital Land Classification Data

**DOI:** 10.3390/s18092925

**Published:** 2018-09-03

**Authors:** Hai Li, Jie Wang, Yi Fan, Jungong Han

**Affiliations:** 1 Tianjin Key Lab for Advanced Signal Processing, Civil Aviation University of China, Tianjin 300300, China; haili@cauc.edu.cn (H.L.); 9215wangjie@163.com (J.W.); yifan@cauc.edu.cn (Y.F.); 2 School of Computing & Communications, Lancaster University, Lancaster LA1 4YW, UK

**Keywords:** high-fidelity, inhomogeneous, ground clutter, DEM, DLCD

## Abstract

This paper presents a high-fidelity inhomogeneous ground clutter simulation method for airborne phased array Pulse Doppler (PD) radar aided by a digital elevation model (DEM) and digital land classification data (DLCD). The method starts by extracting the basic geographic information of the Earth’s surface scattering points from the DEM data, then reads the Earth’s surface classification codes of Earth’s surface scattering points according to the DLCD. After determining the landform types, different backscattering coefficient models are selected to calculate the backscattering coefficient of each Earth surface scattering point. Finally, the high-fidelity inhomogeneous ground clutter simulation of airborne phased array PD radar is realized based on the Ward model. The simulation results show that the classifications of landform types obtained by the proposed method are more abundant, and the ground clutter simulated by different backscattering coefficient models is more real and effective.

## 1. Introduction

Compared with ground-based radars, airborne radars have good fields of vision and mobility, strong survivability, and can find ultra-low-flying targets and ground moving targets [1]. When the airborne radar operates in the down-look mode, the useful signal will be disrupted by strong clutter. Therefore, the clutter suppression will directly affect the performance of the target detection and identification. In Electronic Intelligence (ELINT) systems, researchers have proposed many novel and meaningful methods for target detection and recognition [2,3,4,5,6]. However, the current clutter suppression of airborne phased array Pulse Doppler (PD) radar mainly uses adaptive processing technology [7], where the adaptive processing technique based on the homogeneous clutter needs to meet the Reed, Mallett, Brennan (RMB) criterion [7] to suppress ground clutter. However, when the airborne radar operates in an inhomogeneous clutter environment, it is impossible to obtain sufficiently independent and identically distributed (IID) training samples to estimate the clutter covariance matrix, which adversely affects the clutter suppression performance of the airborne radar. In this case, the Defense Advanced Research Projects Agency (DARPA) launched in 2002 “Knowledge Aided Sensor Signal Processing and Expert Reasoning (KASSPER)”, which studies the use of a priori geographic information to improve the performance of radar target detection and parameter estimation [8]. However, in actual measurement, obtaining real ground clutter data with abundant prior information always comes at the costs of lots of manpower and material resources. Therefore, it is very important to construct a high-fidelity inhomogeneous ground clutter model that complies with the real geographical environment to improve the target detection and parameter estimation performance of the airborne phased array PD radar in inhomogeneous ground clutter environment.

Ground clutter simulation can be divided into ground clutter simulation based on statistical models and ground clutter simulation aided by geographic information according to whether the actual climatic information is involved in the ground clutter simulation [9]. The ground clutter simulation based on a statistical model assumes that the radar area is a plane, and does not consider the actual complex and changeable landform and geomorphic features so that it is often used to achieve a homogeneous clutter simulation meeting a certain probability distribution. Differently, ground clutter simulation aided by geographic information calculates backscattering coefficients of clutter units using actual ground scene information (such as DEM data, DLCD, etc.), which is good at simulating inhomogeneous clutter and can provide effective a priori geographic information for airborne phased array PD radar to further verify the performance of the ground clutter suppression algorithm.

Researchers have carried out a large number of studies on inhomogeneous clutter modeling aided by geographic information. In [10], authors provide a ground clutter simulation method for airborne PD radar under natural scenes. This method gives the shield processing for each unit based on DEM data, and use the echo signal model of PD radar to simulate ground clutter. Hellard et al. [11] provides clutter power maps according to digital terrain relief and landcover information, but the ground clutter simulation is developed for ground-based radars. Alternatively, Kurekin et al. [12] proposes a new method for generating a specific clutter map of ground-based radars using multi-mode remote-sensing images and digital terrain data. This method builds a backscattering coefficient model based on ground-based radars and is not suitable to describe the clutter characteristics of airborne radar. Aiming at airborne cognitive radar, Rao et al. [13] proposes a ground clutter modeling method for airborne cognitive radar based on DEM data. This method takes advantage of DEM data to extract topographical factors and construct the ground clutter in combination with geomorphology theory, which has certain practical values. The above methods apply the priori geographic information to the ground clutter simulation and obtain a more realistic ground clutter simulation result. However, the backscattering coefficient models adopted by the above methods are relatively simple, and only the terrain factors are calculated from the DEM data to determine the type of the landform. Therefore, the data resolution is not high, the landform classification is rough, and the inhomogeneous real terrain cannot be high-fidelity Fitting.

In view of the above problems, this paper presents a high-fidelity inhomogeneous ground clutter simulation method for airborne phased array PD radar aided by DEM and DLCD. Our method combines DLCD with DEM data, and calculates the backscattering coefficients based on matching the different backscattering models with the landform type information in DLCD such that the high-fidelity inhomogeneous ground clutter simulation of airborne phased array PD radar is implemented with Ward ground clutter model. Owing to the use of DLCD in our method, the classifications of the landform types are enhanced, and the classifications of different backscattering coefficient models based on the landform types greatly improve the accuracy and authenticity of the simulated ground clutter.

## 2. Ground Clutter Modeling Aided by Geographic Information

**Figure 1:**
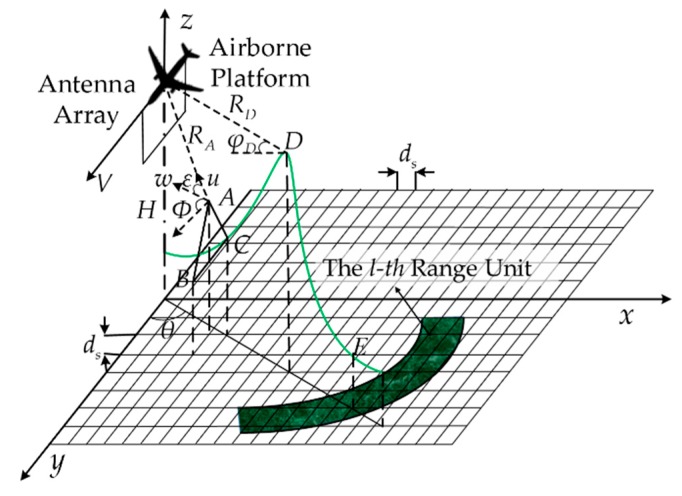
Ground clutter model of airborne phased array PD radar.

Figure 1 shows the ground clutter model of airborne phased array PD radar. In contrast to the other methods, our simulation method taking both DEM and DLCD into account can effectively obtain the coverage of the surface classification, and match the suitable backscattering coefficient models at the same time to classify the landform types and calculate the backscattering coefficients more accurately. The keys of the ground clutter simulation method proposed in this paper lie in the information matching between DEM data and DLCD and the classification matching of backscattering coefficient models, which will be discussed separately below.

### 2.1. DEM Data Information Extraction and Processing

DEM is the discrete model of the actual terrain spatial distribution features, mainly describing the elevation information of the actual terrain. DEM data uses equal-interval square grids to represent the elevation values of the surface grid points, and stores the data in the form of a matrix. By setting the coordinates of the aircraft position, the geographic information of each grid point (i.e., the surface scattering point) can be extracted from the data.

#### 2.1.1. DEM Data Information Extraction

In the inhomogeneous clutter simulation, the information that needs to be extracted from the DEM data mainly includes elevation values, slant range values, azimuth angles, pitch angles, and grazing angles of surface scattering points in various places. As shown in Figure 1, the position of the aircraft is set as $\left(x_{p},y_{p},H\right)$, and the slant range, azimuth angle and pitch angle of any surface scattering point m in the DEM data with respect to the aircraft are respectively calculated by the following formula [14]:

(1) $$\left\{ \begin{array}{l} R_{m}=\sqrt{{(x_{p}-x_{m}),}^{2}+{\left(y_{p}-y_{m}\right)}^{2}+{\left(H-H_{m}\right)}^{2}}\\ \theta_{m}=arctan(x_{m}/y_{m})\\ \varphi_{m}=arcsin((H-H_{m})/R_{m}) \end{array}\right.$$

where $\left(x_{m},y_{m},H_{m}\right)$ is the coordinate of the surface scattering point m. $H_{m}$ is the elevation value of the surface scattering point m, which can be directly obtained by the selected DEM data.

Grazing angle [9] is the angle between the connecting line of the airborne radar and the surface scattering point and the plane where the surface scattering point is located. When calculating the backscattering coefficient, the size of the grazing angle must be determined. In the homogeneous clutter simulation, the grazing angle is the pitch angle of the surface scattering point. However, when the airborne radar is operating in the complex and fluctuating landform, the grazing angle is not equal to the pitch angle of the scattering point. In this case, the calculation of the grazing angle has to start by calculating the sight vector of the surface scattering point to the aircraft followed with the calculation of the normal vector of the plane where the surface scattering point is located. In the end, the grazing angle of each surface scattering point is determined according to the relationship between the two vectors.

As shown in Figure 1, $A(x_{a},y_{a},H_{a})$, $B(x_{b},y_{b},H_{b})$, $C(x_{c},y_{c},H_{c})$ are three adjacent surface scatter points in the DEM data, which form a real terrain surface (when the resolution of the data is high, the space between the grids is small, and the surface is considered as a plane). For the surface scattering point A to be measured, the grazing angle can be obtained by:

(2) $$\Phi_{A}=\pi /2-arccos(w\cdot u/(\|w\|,\left\|u\right\|))$$

where w is the normal vector of the triangle plane where point A is located, u is the sight vector from point A to the aircraft.

For the calculation of w, $\vec{AB}$ and $\vec{AC}$ can be obtained by Figure 1:

(3) $$\begin{array}{l} \vec{AB}=(x_{b}-x_{a},y_{b}-y_{a},H_{b}-H_{a}).\\ \vec{AC}=(x_{c}-x_{a},y_{c}-y_{a},H_{c}-H_{a}) \end{array}$$

Assuming vector w=(x,y,z), since w is the normal vector of the plane where point A is located, w is vertical to $\vec{AB}$ and $\vec{AC}$, then:

(4) $$\left\{ \begin{array}{l} x(x_{b}-x_{a})+y(y_{b}-y_{a})+z(H_{b}-H_{a})=0\\ x(x_{c}-x_{a})+y(y_{c}-y_{a})+z(H_{c}-H_{a})=0 \end{array}.\right.$$

In order to ensure that w points to the positive Z axis, take z=1. Solution of the above equations can be obtained by:

(5) $$w=((H_{a}-H_{b})/d_{s},(H_{a}-H_{c})/d_{s},1),$$

where $d_{s}$ is the resolution of the data (i.e., grid spacing).

For the calculation of u, according to the location of the aircraft can get the sight vector of point *A*:

(6) $$u=(x_{p}-x_{a},y_{p}-y_{a},H-H_{a}),$$

and so on, making it is possible to obtain the grazing angles of all surface scattering points within the operating range of radar.

#### 2.1.2. Shield Processing

When the airborne PD radar operates in the down-look mode, there will be surface scattering points that cannot be irradiated due to the undulating landform within the operating range of the radar [9,15]. Since these scattering points do not contribute to clutter simulation, in the ground clutter simulation, we must consider the shield processing, where the shields mainly include sight-shield and self-shield. Here, we will introduce the causes of these two kinds of shield and the processing flow respectively.

As shown in Figure 1, taking two surface scattering points D and E in the same direction of airborne radar as an example, the shield processing will be described in detail. Assuming that E is the surface scattering point to be measured. Based on the DEM data, the slant ranges of the two scattering points relative to the aircraft are respectively $R_{D}$ and $R_{E}$, $\Phi_{D}$ and $\Phi_{E}$ are grazing angles respectively, then:

- If $R_{D}<R_{E}$ and $\Phi_{D}<\Phi_{E}$, then the surface scattering point E at low elevation is shielded by the surface scattering point D at high altitude, and the surface scattering point E cannot be irradiated by the radar (i.e., the point E is not visible to the radar), this situation is the sight-shield;
- If the angle between the normal vector of the plane where the surface scattering point E is located and the sight vector is greater than 90° (i.e., $\Phi_{E}$ is less than 0°), this situation is self-shield.

When there is a sight-shield or a self-shield on the surface scattering point E to be measured, $\Phi_{E}$ is set to be 0° to complete the shield processing on the surface scattering point E.

### 2.2. Matching and Calculation of Backscattering Coefficient Model Based on DLCD

The backscattering coefficient model is a model of the relationship between the backscatter coefficient and various influencing factors (including grazing angle, geomorphology, radar operating frequency, etc.). So far, researchers have proposed several backscattering coefficient models related to landform, including the Morchin model [16], the modified Morchin model [17], the F.T. Ulaby model [18] and the γ-*f* model [19]. Based on these models, combined with DEM data, inhomogeneous ground clutter simulation can be achieved. However, the classification of landform types is only based on the calculation of terrain factors by DEM data, the classification of landforms are relatively rough, and the single backscattering coefficient model cannot exactly match the inhomogeneous actual terrain. In view of this, this paper uses the abundant landform classification information in DLCD to match different backscattering coefficient models to realize high-fidelity inhomogeneous ground clutter simulation.

Different backscattering coefficient models match the different types of landform. The modified Morchin model can well match the natural landform of the land surface, while the Morchin model only matches with the water surface landform. The γ−f model is used to model the urban landform, and it can be well applied to the simulation of the urban clutter. Therefore, in this paper, aiming at the different types of landform in DLCD, the modified Morchin model, Morchin model and γ−f model are respectively used to simulate the clutter of different landform types. Mathematically matching water surface landform, natural landform of the land surface, and urban landform models and their backscattering coefficients are discussed here. 

#### 2.2.1. Backscattering Coefficient Calculation of Water Surface Landform

The water surface landform is the more common type of landform in the actual work of airborne PD radar and the type of landform clearly marked in DLCD. The Morchin model can well match water surface landform to get more accurate backscattering coefficient of water surface landform, and the water surface backscattering coefficient at the m-th(m=1,2,⋯,M) surface scattering point can be calculated by the following formula:

(7) $$\sigma_{Wa,}(m)=\frac{4\times 10^{-7}\times 10^{0.6(ss+1)}\sigma_{c}sin(\Phi_{m})}{\lambda }+cot^{2}(\beta )exp[-\frac{tan^{2}(\pi /2-\Phi_{m})}{tan^{2}(\beta )}]$$

where M is the number of scattering points in the simulation area; $\Phi_{m}$ is the grazing angle; ss is the sea level (that is, the fluctuation of the water surface); $\beta =[2.44{\left(ss+1\right)}^{1.08}]/57.29$, the unit is rad:

(8) $$\sigma_{c}=\left\{ \begin{array}{ll} {\left(\frac{\Phi_{m},}{\Phi_{c}}\right)}^{k} & \Phi_{m}<\Phi_{c}\\ 1 & \Phi_{m}>\Phi_{c} \end{array}\right.$$

where $\Phi_{c}=arcsin(\lambda /4\pi h_{e})$, the unit is rad; $h_{e}\approx 0.025+0.046ss^{1.72}$, the unit is meter, represents the roughness of the water surface; Recommend k=1.9. It can be seen from the above, the grazing angle and the sea level series are the keys to calculate the water surface backscattering coefficient.

#### 2.2.2. Backscattering Coefficients Calculation of Different Land Surface Natural Landforms

In high-resolution DLCD, the natural landform is carefully subdivided into various types of landform, and detailed coding of each landform type has been made. In the above models of backscattering coefficients, the modified Morchin model is more land-based and thus suitable for the landforms given in the DLCD. Therefore, in this paper, the modified Morchin model is selected based on the abundant landform types in DLCD to calculate the backscattering coefficient of each land surface natural landform, and then, the land surface backscattering coefficient of the m-th(m=1,2,⋯,M) surface scattering point can be calculated by the following formula:

(9) $$\sigma_{La_{x},}(m)=\frac{\rho \sigma_{c}sin(\Phi_{m})}{\lambda }+u_{0}cot^{2}(\beta_{0})exp[-\frac{tan^{2}(\chi -\Phi_{m})}{tan^{2}(\beta_{0})}]$$

where M is the number of scattering points in the simulation area; $\Phi_{m}$ is the grazing angle; $u_{0}=\sqrt{f}/4.7$, f is the radar operating frequency, the unit is GHz; The subscript $La_{x}(x=1,2,3,\cdots )$ represents the type of natural landform of each land surface divided in the DLCD, and $\sigma_{c}$, ρ, χ, $\beta_{0}$ are constants determined by the landform types as shown in Table 1 [9].

#### 2.2.3. Backscattering Coefficient Calculation of Urban Landform

In the high-resolution DLCD, man-made landform types, such as cities, are clearly identified by surface classification codes. Therefore, it is necessary to determine the appropriate backscattering coefficient model to fit the urban landform, and then accurately calculate the backscattering coefficient of the urban landform. γ-f model defines the backscattering coefficient of urban landform, which can be well used in urban clutter simulation. And then, the urban backscattering coefficient of the m-th(m=1,2,⋯,M) surface scattering point can be calculated by the following formula:

(10) $$\sigma_{Ci,}(m)=\gamma af^{b}sin(\Phi_{m}+c)$$

where γ, a, b, c are the parameters related to the types of landform. For the urban landform, γ=0.316, a=0.36, b=0.18, c=0.7; f is the radar operating frequency, the unit is GHz.

### 2.3. Ground Clutter Simulation of Airborne Phased Array PD Radar Based on Ward Model

**Figure 2:**
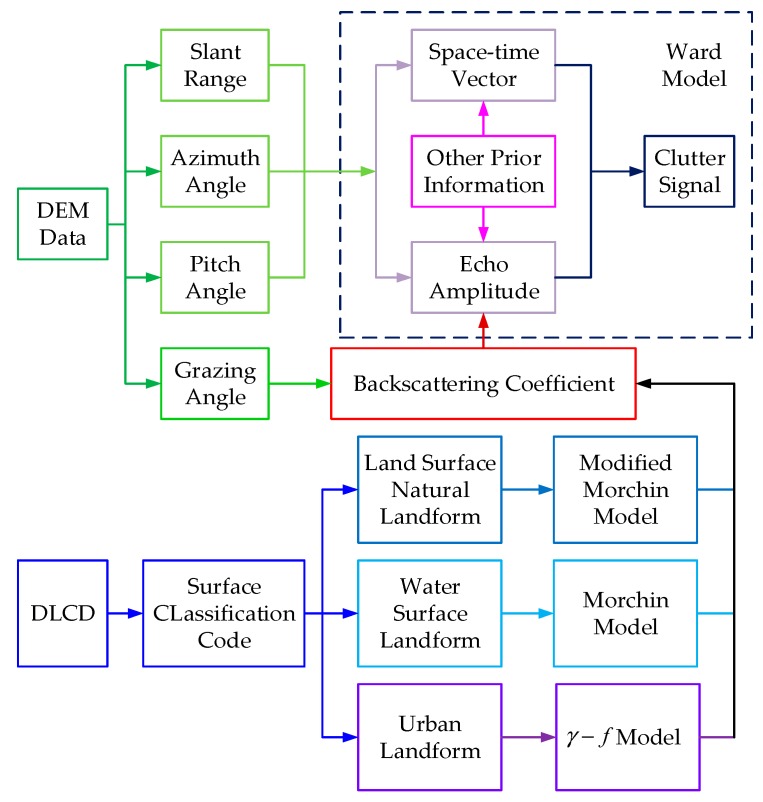
Flow chart of geographic information processing based on DEM and DLCD.

Figure 2 shows the flow chart of geographic information processing based on DEM and DLCD. It can be seen from the figure that the slant range, azimuth angle and pitch angle of each surface scattering point relative to the aircraft determined by the DEM data and the backscattering coefficient $\sigma_{q}(m)$ (the subscript $q=Wa,La_{x}\;or\;Ci$ represents the type of landform corresponding to the *m*-th scattering point in the DLCD) calculated from the backscattering coefficient model matched by the surface code of DLCD are the keys to calculate the echo intensity of scattering points. 

Assuming that the aircraft speed is uniform and straight, and the speed is V, the N elements line array is placed uniformly on the airborne platform along the heading direction. The element interval is d=0.5λ, λ is the radar wavelength, and the pulse repetition frequency is $f_{r}$. And assuming that the *l*-th range unit contains M surface scattering points. According to the Ward [20] model, the data of the *m*-th scattering point of the range unit under the *k*-th pulse sampling of the *n*-th radar element can be expressed as:

(11) $$\begin{array}{ll} c_{nklm,} & =\alpha_{lm}e^{j(n-1)\omega_{s,lm}+j(k-1)\omega_{t,lm}}\\ & =\sqrt{\frac{P_{t}G_{t}g_{t}\lambda^{2}\sigma_{q}(m)d_{s}^{2}}{{\left(4\pi \right)}^{3}R_{m}^{4}L_{s}}}F(\theta_{m},\varphi_{m})e^{j(n-1)\omega_{s,lm}+j(k-1)\omega_{t,lm}} \end{array}$$

where $\alpha_{lm}$ represents the echo intensity of the *m*-th surface scattering point in the *l*-th range unit, $P_{t}$ is the peak power of the radar, $G_{t}$ is the gain of the array antenna, $g_{t}$ is the receive gain of the array antenna, $L_{s}$ is the system loss, $F(\theta_{m},\varphi_{m})$ is the antenna pattern, which can be expressed as:

(12) $$F(\theta_{m},\varphi_{m})=\sum_{n=1,}^{N}I_{n}e^{j\frac{2\pi d}{\lambda }(n-1)(cos\theta_{m}cos\varphi_{m}-cos\theta_{0}cos\varphi_{0})}$$

here $\theta_{m}$ is the azimuth angle of the *m*-th surface scattering point in the *l*-th range unit, $\varphi_{m}$ is the pitch angle of the *m*-th surface scattering point in the *l*-th range unit, $I_{n}$ is the weight of the sub-array, $\theta_{0}$ and $\varphi_{0}$ represent the antenna main lobe azimuth and pitch angle. In Formula (11), $\omega_{s,lm}$ and $\omega_{t,lm}$ respectively represent the spatial angular frequency and the temporal frequency of the *m*-th surface scattering point in the *l*-th range unit, which can be expressed as:

(13) $$\left\{ \begin{array}{l} \omega_{s,lm.}=\frac{2\pi d}{\lambda }cos\theta_{m}cos\varphi_{m}\\ \omega_{t,lm}=\frac{4\pi V}{f_{r}\lambda }sin\theta_{m}cos\varphi_{m} \end{array}\right.$$

By adding the echo sample data of all the surface scattering points in the *l*-th range unit, the total clutter of the *l*-th range unit received by the *k*-th pulse of the *n*-th antenna element of the radar can be obtained by:

(14) $$C_{l}(n,k)=\sum_{m=1.}^{M}\alpha_{lm}e^{j(n-1)\omega_{s,lm}+j(k-1)\omega_{t,lm}}$$

By arranging the received clutter sampling data $C_{l}$ into a column vector, the space-time snapshot data $c_{l}$ in the *l*-th range unit can be obtained by:

(15) $$\begin{array}{l} c_{l}=span(C_{l})\\ ={\left[\underset{k=1}{\underset{︸}{\begin{array}{cccc} C_{l}(1,1). & C_{l}(2,1) & \cdots & C_{l}(N,1) \end{array}}}\begin{array}{c} \cdots \end{array}\underset{k=K}{\underset{︸}{\begin{array}{cccc} C_{l}(1,K) & C_{l}(2,K) & \cdots & C_{l}(N,K) \end{array}}}\right]}_{NK\times 1}^{T} \end{array}$$

The power spectrum of the space-time two-dimensional clutter from the *m*-th surface scattering point can be obtained as [21]:

(16) $$P_{m}=\frac{1}{A_{m}^{H}(s,t)RR_{c}^{-1,}A_{m}(s,t)}$$

where $RR_{c}=C_{c}\times C_{c}{}^{H}$ represents clutter covariance matrix, $C_{c}={\left[c_{1}c_{2}\cdots c_{L}\right]}_{NK\times L}$. The space-time steering vector for *m*-th surface scattering point is $A_{m}(s,t)=a_{t}(V)⊗a_{s}(\theta_{m},\varphi_{m})$, $a_{t}(V)$ and $a_{s}(\theta_{m},\varphi_{m})$ respectively represent the time and space steering vectors:

(17) $$\begin{array}{l} a_{t}(V)={\left[1e^{j\pi \frac{4V.}{\lambda f_{r}}cos\theta_{m}cos\varphi_{m}}\cdots e^{j\pi (K-1)\frac{4V}{\lambda f_{r}}cos\theta_{m}cos\varphi_{m}}\right]}^{T}\\ a_{s}(\theta_{m},\varphi_{m})={\left[1e^{j\frac{2\pi d}{\lambda }cos\theta_{m}cos\varphi_{m}}\cdots e^{j\frac{2\pi d}{\lambda }(N-1)cos\theta_{m}cos\varphi_{m}}\right]}^{T} \end{array}$$

## 3. Simulation Flow

**Figure 3:**
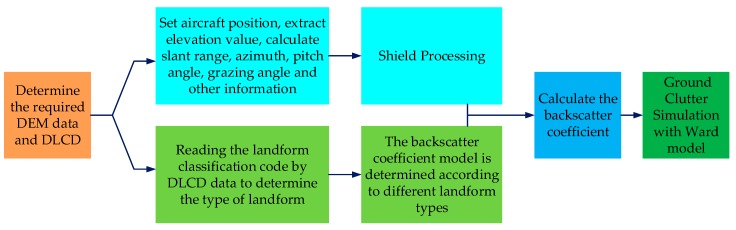
Flow chart of high-fidelity inhomogeneous ground clutter simulation.

Figure 3 is the flow chart of high-fidelity inhomogeneous ground clutter simulation. From the figure, the key steps of the high-fidelity inhomogeneous clutter simulation method for airborne phased array PD radar aided by DEM and DLCD are:

*Step 1*. Determine the required DEM [22] and DLCD data [23];
*Step 2*. Calculate the elevation value, the slant range, azimuth, pitch angle, and grazing angle of each surface scattering point relative to the aircraft from DEM data;
*Step 3*. Shield processing according to the grazing angle and slant range, and remove surface scattering points that do not contribute to the radar echo;
*Step 4*. Read the land surface classification codes of the surface scattering points, determine the types of different landforms and match different backscattering coefficient models;
*Step 5*. Calculate the backscattering coefficients of the scattering points at various locations within the radar range and use Ward model to realize high-fidelity inhomogeneous ground clutter simulation.

## 4. Simulation Results Analysis and Discussion

### 4.1. Simulation Condition Description

In this paper, the aircraft and radar simulation parameters are shown in Table 2.

### 4.2. Simulation Results

**Figure 4:**
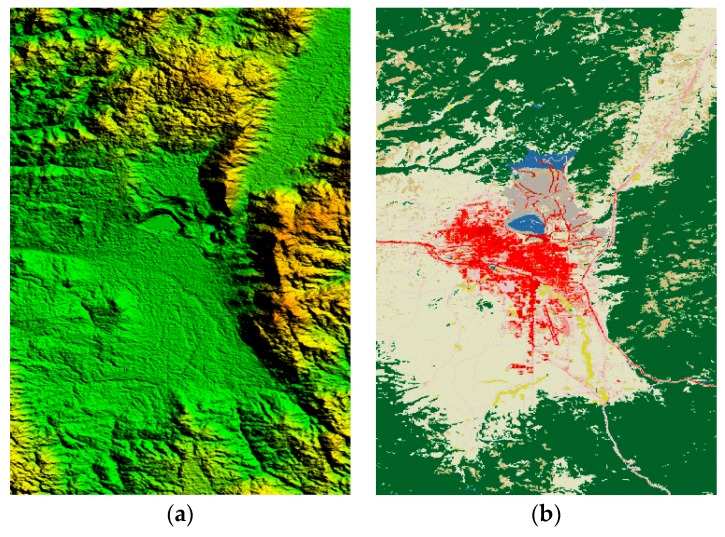
The original maps of the DEM data and DLCD: (**a**) The original maps of the DEM data; (**b**) The original maps of the DLCD.

Figure 4a,b are the original maps of the DEM data and DLCD used in the simulation presented here, ranging from latitude 45°51′ N to 46°09′ N and longitude 112°24′ W to 112°36′ W. As can be seen from the DLCD map, the selected area includes cities (red), high mountains (green), water surface (blue) and scrub (brown), which are very universal.

**Figure 5:**
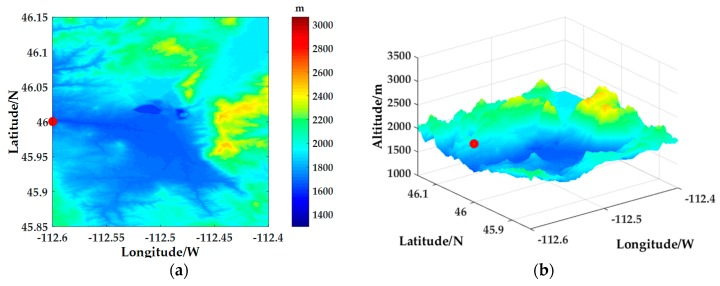
Aircraft position setting and elevation value display in radar operating range: (**a**) Two dimensional elevation value display; (**b**) 3D elevation value display.

**Figure 6:**
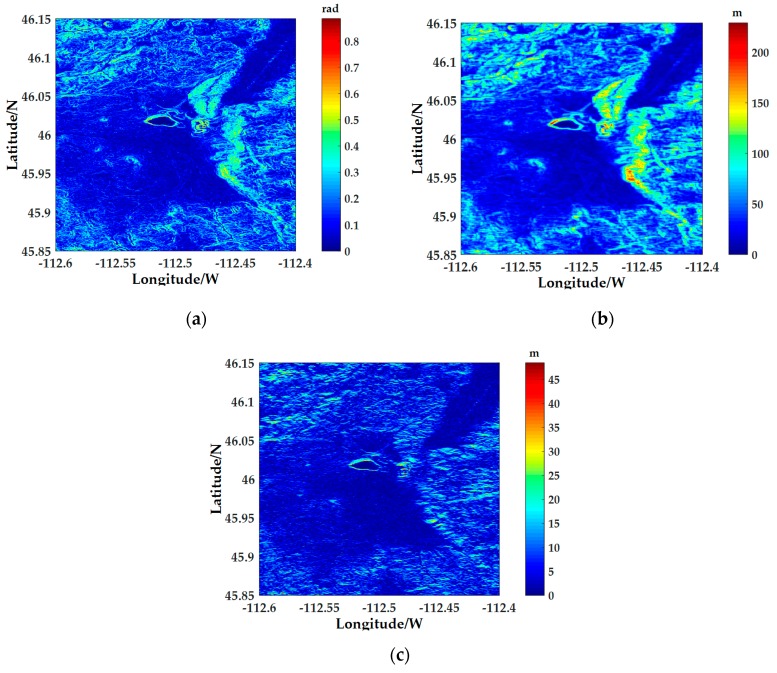
Topographic factors of the simulation area: (**a**) The slope of the simulation area; (**b**) The terrain relief of the simulation area; (**c**) The surface roughness of the simulation area.

The aircraft (marked with a red dot in the figure) is set at 46° N and 112°36′ W and flies from west to east. The DEM in the radar operating range is shown in Figure 5. And the slope, terrain relief and surface roughness calculated from the DEM data in the area are shown in Figure 6a–c, respectively. It can be seen from Figure 5 and Figure 6 that the selected simulation area is complex and the terrain is drastically undulating, especially in the water-land boundary and the mountain terrain, the slope changes greatly, which results in significant inhomogeneous characteristics of ground clutter in the simulation region.

**Figure 7:**
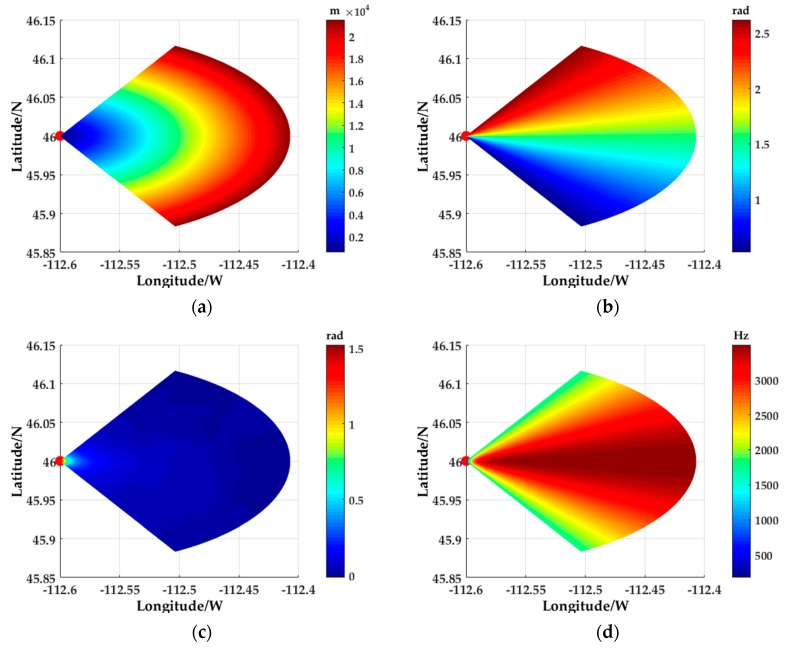
Geographical information of surface scattering points: (**a**) The slant rang of surface scattering points; (**b**) The Azimuth of surface scattering points; (**c**) The pitch angle of surface scattering points; (**d**) The Doppler information of surface scattering points.

The slant ranges, azimuth angles, pitch angles, and Doppler information of ground scattering points relative to the Aircraft within the radar operating range are shown in Figure 7a–d. It can be clearly seen from these figures that the proposed method can effectively extract the slant range, azimuth angle, pitch angle, and Doppler information of each surface scattering point from the DEM data, especially the change of pitch angle to the terrain is particularly acute. This shows that the inhomogeneous characteristics of ground clutter is very obvious.

**Figure 8:**
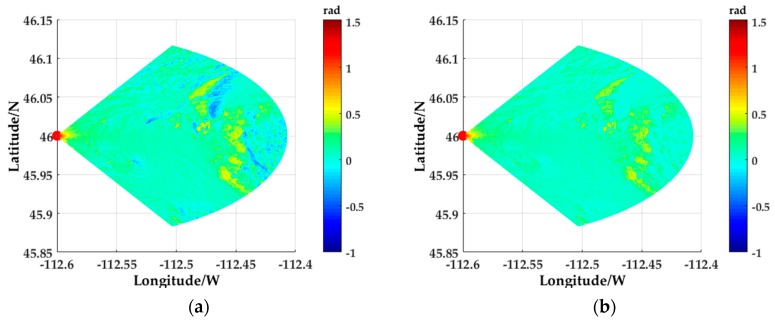
Grazing angle calculation and shield processing: (**a**) The grazing angle of the surface scattering point in the radar operating range; (**b**) The grazing angle after the shield processing.

Figure 8a shows the grazing angles of the surface scattering points in the radar operating range, and Figure 8b shows the grazing angles after the shield processing. As can be seen from the comparison between the two figures, there are a large number of surface scattering points in the selected terrain which are sight-shield and self-shield. Especially in the vicinity of the mountain, there are obvious sight-shield that do not contribute to the simulation of clutter.

**Figure 9:**
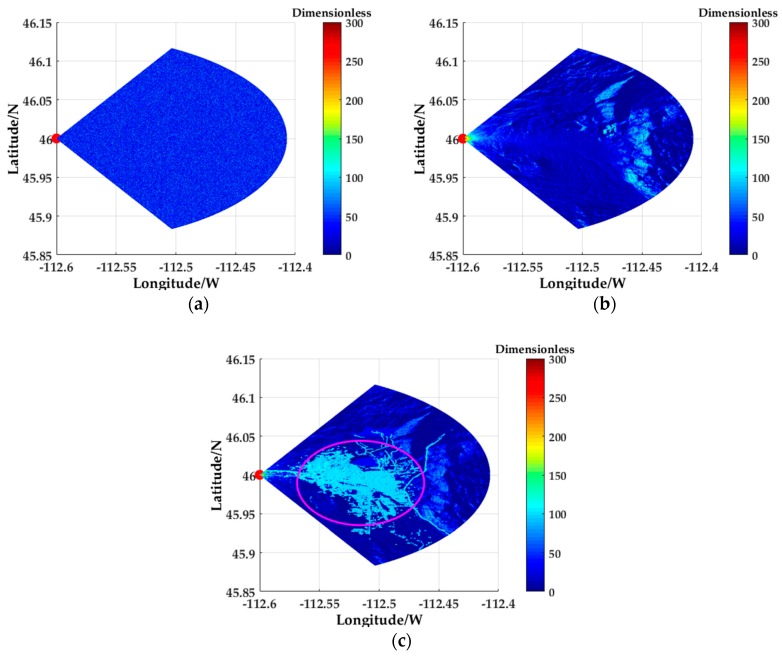
Comparison of backscattering coefficients in different methods: (**a**) Gaussian distribution backscattering coefficients based on a traditional statistical model; (**b**) The backscattering coefficients obtained by using DEM data proposed in [13]; (**c**) The backscattering coefficients based on the method proposed in this paper.

**Figure 10:**
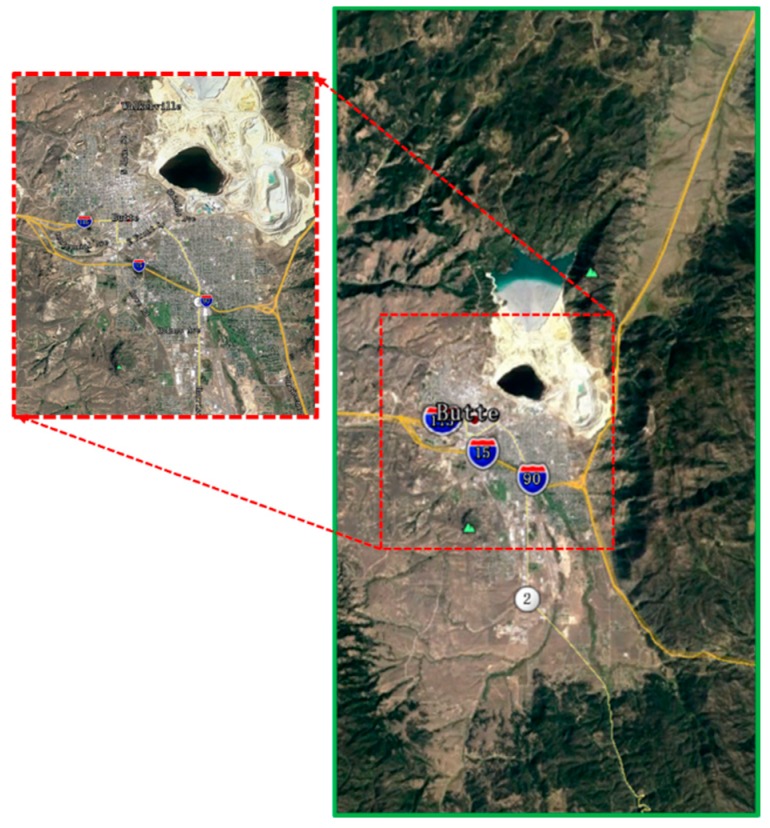
The satellite map of the Butte area in Google Maps.

Figure 9 shows the backscattering coefficients obtained by different methods. Figure 9a is a Gaussian distribution backscattering coefficient based on a traditional statistical model. Figure 9b is the backscattering coefficient obtained by using DEM data to calculate the topographic factors (including slope, ground curvature, terrain relief and surface roughness) proposed in [13]. As can be seen from Figure 9a,b, the method in Figure 9b shows obvious inhomogeneous characteristics in the backscattering coefficient obtained by calculating the topographical factors. Figure 9c is the backscattering coefficient based on the method proposed in this paper. It is obtained by matching the backscattering coefficient model of DLCD and calculating the grazing angles based on the DEM data. The circled area is the Butte area, USA. Figure 10 is a satellite map of the Butte area in Google Maps. After comparison, it can be clearly seen that the backscattering coefficient calculated by the proposed method can better match the actual terrain.

**Figure 11:**
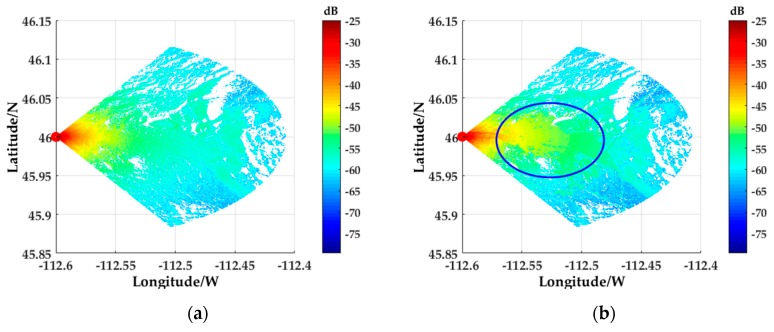
Comparison of clutter power spectrum: (**a**) The clutter power spectrum obtained by using only DEM data proposed in [13]; (**b**) The clutter power spectrum obtained by the method proposed in this paper.

Figure 11 is a comparison chart of clutter power spectrum, in which Figure 11a,b is the clutter power spectrum obtained by using only DEM data proposed in [13] and the clutter power spectrum obtained by the method proposed in this paper. By comparing the two graphs, it is clear that the proposed method can be used to simulate ground clutter with complex landform features due to the introduction of DLCD, especially for urban clutter (the echo power spectrum of the US Butte area in the blue circle). Additionally, it seems that using only DEM data cannot well simulate ground clutter with complex landform features.

**Figure 12:**
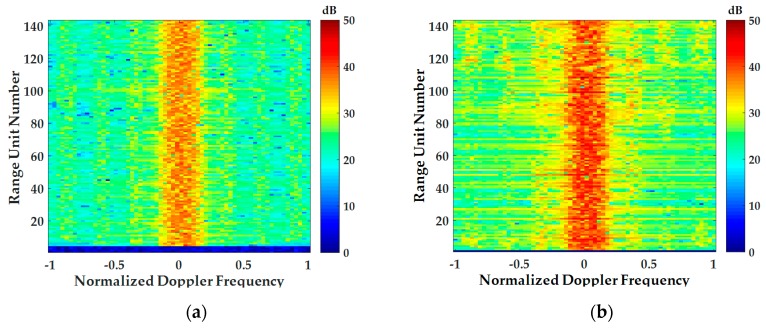
Comparison of range Doppler spectrum: (**a**) The range Doppler spectrum obtained from the Gaussian statistical model homogeneous clutter simulation method; (**b**) The high-fidelity inhomogeneous clutter simulation method proposed in this paper.

Figure 12 shows the comparison of the range Doppler spectrum obtained from the Gaussian statistical model homogeneous clutter simulation method and the high-fidelity inhomogeneous clutter simulation method proposed in this paper. Figure 12a shows the range Doppler spectrum of homogeneous clutter, and Figure 12b shows the range Doppler spectrum of the method proposed in this paper. After comparison, it can be seen that the ground clutter simulated by using DEM and DLCD has obvious inhomogeneous characteristics with the change of the range unit.

**Figure 13:**
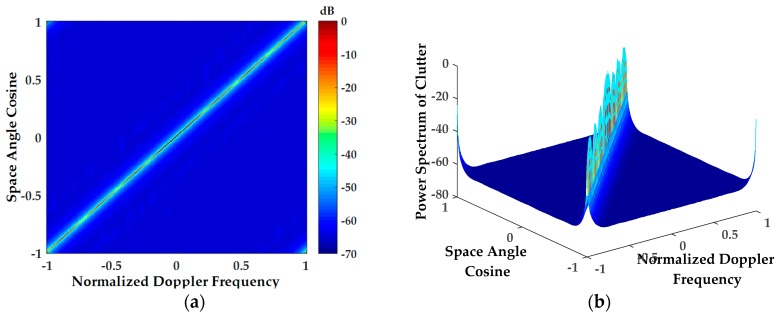
Space-time spectrum of ground clutter: (**a**) Two-dimensional space-time spectrum; (**b**) 3D space-time spectrum.

Figure 13 shows the space-time spectrum of ground clutter for airborne phased array PD radar. It can be seen from the figure that due to the frequency expansion caused by the aircraft motion, the space-time spectrum of the ground clutter of an airborne side-looking PD radar is diagonally distributed, which is the main feature of airborne phased array PD radar side-looking clutter.

## 5. Conclusions

In order to verify the performance of the airborne PD radar clutter suppression algorithm in a simulated computational environment, this paper proposes an airborne phased array PD radar high-fidelity inhomogeneous ground clutter simulation method aided by DEM and DLCD, which can provide high-fidelity inhomogeneous clutter data for airborne phased array PD radar signal processing. This method obtains altitude, azimuth angle, pitch angle, grazing angle and other information of the terrain unit according to the DEM data of the selected latitude and longitude, and judges the terrain shielding. According to the DLCD, the earth’s surface classification codes are read, the landform types are determined, and different backscatter coefficient models are matched, and then the backscattering coefficient is calculated by combining the geographic information extracted by DEM data and the backscattering coefficient model matched by DLCD. Finally, the high-fidelity inhomogeneous clutter simulation of airborne phased array PD radar is realized with the Ward ground clutter model. The simulation results show that the clutter simulation method proposed in this paper uses high-resolution data, and the classifications of landform types are rich and detailed. Different backscattering coefficient models are selected based on landform type classification to make the simulated ground clutter more real and effective. However, due to the relatively large working range of the radar and the high resolution of the data, the computational burden of the method is relatively heavy, and the timeliness needs to be improved.

## Figures and Tables

**Table 1:** Parameter values of landform types.

Code	Landform Types	$\sigma_{c}$	ρ	χ	$\beta_{0}$
$La_{1}$	Farmland	1	0.004	π/2	0.2
$La_{2}$	Hills	1	0.0126	π/2	0.4
$La_{3}$	Mountain	1	0.04	π/2	0.5
$La_{4}$	Mud	1	0.001945	π/2	0.155
$La_{5}$	Snow Land	1	0.00263	π/2	0.17
$La_{6}$	Grassland	1	0.00572	π/2	0.24
$La_{7}$	Crop Land	1	0.00744	π/2	0.28
$La_{8}$	Forest	1	0.00916	π/2	0.32
$La_{9}$	Bare Land	1	0.01088	π/2	0.36

**Table 2:** Parameter values of aircraft and radar.

Aircraft Height (m)	600	Element Number	8
Aircraft Speed (m/s)	87.5	Sample Pulse Number	64
Radar Wavelength (m)	0.05	Main Lobe Direction (°)	(90, 15)
Pulse Repetition Frequency (Hz)	7000	CNR (dB)	50

Note: In the simulation experiment, we set the azimuth angle of the main lobe of the radar to be 90° and the pitch angle to be 15°. That is to say, the main beam of the radar is perpendicular to the antenna array and has an angle of 15° with the horizontal ground.
